# Thermal Conductivity of Protein-Based Materials: A Review

**DOI:** 10.3390/polym11030456

**Published:** 2019-03-11

**Authors:** Ye Xue, Samuel Lofland, Xiao Hu

**Affiliations:** 1 Department of Physics and Astronomy, Rowan University, Glassboro, NJ 08028, USA; xuey5@rowan.edu (Y.X.); lofland@rowan.edu (S.L.); 2 Department of Biomedical Engineering, Rowan University, Glassboro, NJ 08028, USA; 3 Department of Molecular and Cellular Biosciences, Rowan University, Glassboro, NJ 08028, USA

**Keywords:** thermal conductivity, protein, crystal structure, green materials

## Abstract

Fibrous proteins such as silks have been used as textile and biomedical materials for decades due to their natural abundance, high flexibility, biocompatibility, and excellent mechanical properties. In addition, they also can avoid many problems related to traditional materials such as toxic chemical residues or brittleness. With the fast development of cutting-edge flexible materials and bioelectronics processing technologies, the market for biocompatible materials with extremely high or low thermal conductivity is growing rapidly. The thermal conductivity of protein films, which is usually on the order of 0.1 W/m·K, can be rather tunable as the value for stretched protein fibers can be substantially larger, outperforming that of many synthetic polymer materials. These findings indicate that the thermal conductivity and the heat transfer direction of protein-based materials can be finely controlled by manipulating their nano-scale structures. This review will focus on the structure of different fibrous proteins, such as silks, collagen and keratin, summarizing factors that can influence the thermal conductivity of protein-based materials and the different experimental methods used to measure their heat transfer properties.

## 1. Introduction

Biopolymers are polymers that have components found in nature. They can be synthesized naturally or man-made. Similar to synthetic polymers, biopolymers are long-chain molecules with many repeating units. Based on their main components, biopolymers can be specified into three main categories: proteins (e.g., silks, elastin, resilin, keratin, collagen, and various plant proteins), polysaccharides (e.g., cellulose, starch, and chitin), and nucleic acids (e.g., deoxyribonucleic acid and ribonucleic acid). In addition to biomedical applications, biopolymers, especially protein polymers, are also widely used in green applications, which can significantly reduce or eliminate the use or production of substances hazardous to humans, animals, plants, and the environment [1]. Therefore, protein-based heat transfer materials can be excellent candidates to replace many materials currently used in the market such as synthetic polymers or metals.

Thermal conductivity describes the transport of heat through a material body driven by a temperature gradient. With the rapid development of delicate high-tech instruments, such as ultra large scale integration (ULSI) in digital devices and communication equipment, special materials with tunable thermal conductivity or heat transfer direction are in tremendous demand [2,3]. A better understanding of the thermal conductivity of materials will enhance current material design techniques and applications in various fields. Typically, most polymers are classified as poor conductors, while metals are generally very good conductors. As shown in Table 1, the thermal conductivity of Nylon 6 is 0.25 W/m∙K compared to 400 W/m∙K for copper [4]. The contrasting thermal conductivity values of these two materials in specific, or polymers and metals in general, are caused by their different principles of heat transport [5,6]. In metals, the thermal conductivity can generally be attributed to charge carriers which transfer energy. However, for polymers, heat conduction takes place through lattice vibrations (phonons). In general, the amorphous structure in polymers results in a decrease in the mean free path of phonons, which lowers the material’s thermal conductivity. Moreover, defects in bulk polymers, voids, chain ends, interfaces and impurities also affect a material’s thermal conductivity.

The thermal conductivity of polymers is normally on the order of 0.1 W/m∙K, which makes most polymers good thermal insulators. The insulating properties can be enhanced by foaming them and controlling the pore size in the foams. Today, polymer-based thermal insulation materials have been used in space technology [14], for example, to protect the structural integrity of spaceships. Polymer-based thermal insulation materials are also an important part of buildings, electrical power lines, and clothing for firefighters [15,16,17]. Due to their low density, low thermal expansion and low maintenance, these materials could be utilized in microelectronics, automobiles, and satellite devices as well. On the other hand, with the appropriate nanostructure, polymers can also possess very high thermal conductivity. For example, polymer nanofibers grow in a limited nanotube space have been found to have a thermal conductivity of up to 100 W/m∙K [7] that can be maintained over a wide range of temperature without degradation.

Compared with biopolymers, however, most synthetic polymer materials or metals with highly conducting or insulating properties have obvious drawbacks for certain applications. For instance, thermal transfer films made of polyurethane and polystyrene have a limited temperature usage range because of flammability. In addition, synthetic polymers are often non-biocompatible, which may produce toxic residues when they are used as biomedical materials or food packaging materials. Although metals have high thermal conductivities, they are also electrically conductive and mechanically stiff. 

**Figure 1:**
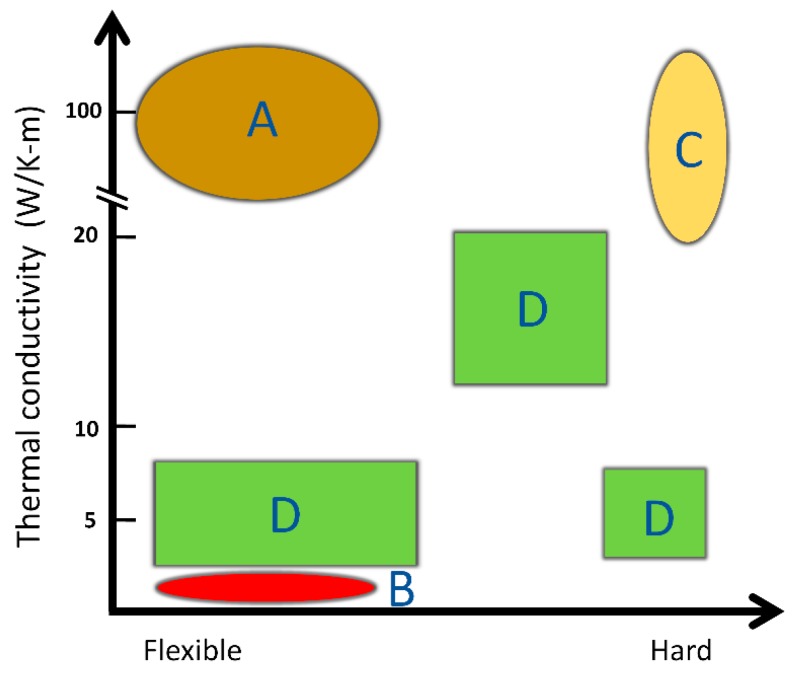
A and B are the ideal areas of thermal conductive and thermal insulating materials using protein-based polymers, respectively; C areas represent metal-based or ceramic-based materials that have both high thermal conductivity and high elastic modulus; D areas represents the typical synthetic polymer-based heat transfer materials that currently exist in the market.

On the other hand, thermal transfer biopolymer materials such as silk, collagen and keratin are mechanically flexible, naturally fire retardant, transparent and biocompatible. Due to their light weight, flexibility, easy processing and corrosion resistance, biopolymer insulators or biopolymer materials with high thermal conductivity have attracted much attention recently [18,19,20]. Protein polymers, such as silk, can be manufactured into diverse applications, such as sensor parts, aerospace recycling components, electrical products, medical materials, and textile materials [21,22,23,24,25]. The relationship between hardness and thermal conductivity in current market is shown in Figure 1. With numerous ongoing studies to increase their thermal conductivity and a tremendous potential market in the future, protein-based thermal conductive materials may have vast application in green and sustainable material industry in the future.

## 2. Structure of Protein-Based Polymers

**Figure 2:**
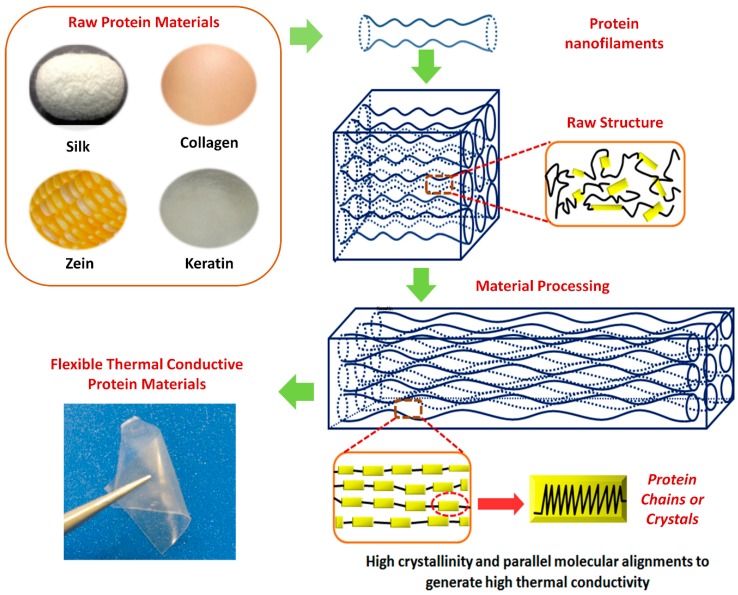
Illustration of the relations between raw protein materials, material processing and flexible thermal conductive protein materials. Tunable thermal conductivity can be achieved through modifying the structure of protein structures.

Protein-based polymers are biocompatible green polymers due to their biological nature and recyclability [26,27]. This covers a broad range of biopolymers such as silks, elastin, keratin, resilin and collagen. They have been used in biomedical fields for many applications because of their marvelous biocompatibility, biodegradability, extraordinary mechanical properties and economic benefits. The properties of natural proteins can be tuned through modifying their structure at micro- or nano-scale. There has been considerable interest as of late to modify the protein structure to achieve high thermal conductivity and Figure 2 shows one pathway that raw protein materials can be processed to reach that goal.

### 2.1. Silk

Silk is a well-known natural fiber produced by silkworms or spiders. Silk has been well studied in the past decades due to its outstanding mechanical durability, stable chemical properties and good biocompatibility [28,29]. It can be classified into wild silk and domestic silk according to the growth environment of the insects. Domestic silkworm silk fiber mainly consists of fibroin and sericin. Silk fibroin accounts for 60–80% of the fiber while sericin accounts for 20–30%. Sericin functions as a natural glue that combines fibroin fibers together [30]. Domestic *Bombyx mori* silk fibroin is characterized with a unique amino acid sequence of GAGAGS, a hydrophobic block which contributes to the formation of β sheets in the fibroin structure [31]. The high tensile strength of silk fiber is attributed to the β sheets while the hydrophobic block contributes to its elasticity [32]. Studies have shown that properties of silk-based materials can be effectively manipulated through controlling the content and alignment of the β sheets [32]. 

Silk fibroin has been manufactured into nanofibers, particles, scaffold and film that can be widely used in biomedical field and healthcare industry [33,34,35]. Regenerated water-based silk fibroin suspension have been coated onto fruits which can effectively modulate the gas diffusion [36] that can help manage fruit freshness during the transportation and in the poor areas where people have no refrigerators. Additionally, silk fibroin has been manufactured into particles as a drug carrier that can realize controllable drug release [34]. Spider silk fiber has been reported with high thermal conductivity, up to 416 W/m∙K, although this claim is not universally accepted [37,38,39].

### 2.2. Collagen

Collagen is a structural protein that mainly exists in the extracellular matrix. Collagen is mostly found in the fibrous connective tissues of animals such as tendons, bones, ligaments, and skin [40]. Arranged collagens provide mechanical support in the connective tissues while fractional collagen provides toughness and maintain the anisotropy for biomineralized material [41,42]. Most of the collagens found in the body are classified into three main types [42], and all collagens share a right-handed triple helix structure [43]. Collagen is called as the “steel of biological materials” and has been extensively investigated [44].

Collagens have been widely used in tissue engineering. It is reported that oriented collagen tubes (OCT) combined with fibroblast growth factor can accelerate the repair of sciatic nerve defects in rat [45]. Large and complex 3D scaffold with uniform and homogeneous porous structure can also been obtained through the freeze-drying method using collagens as raw materials [46].

### 2.3. Keratin

Keratin is a fibrous structural protein that mainly exists in hair, fingernails, scales, feathers and wools [47]. Keratin is known for its excellent chemical stability, and it is insoluble in both water and organic solvents [48]. Keratins can be further classified into type I and type II according to their sequences [49]. They are long and unbranched filaments containing a central alpha helical domain separated by three beta-turn segments [50]. Due to its high molecular diversity, keratin is an important type of intermediate filament. In epithelial cells, keratin filaments are bundled as tonofilaments, which act as bones of the cellular scaffold and contribute rigidity to the cell. They help tissues maintain structural integrity and sustain mechanical stress [50,51].

Good biocompatibility and biodegradability have made keratin one of the most promising biomaterials. Regenerated wool keratin films manufactured from ionic liquid have been well studied. Beta-sheet and alpha-helix structures can be manipulated through changing the process parameters [52,53]. Keratin-PCL nanofibers have been obtained through electrospinning, while cellular compatibility of the composite nanofibers has also been observed [54].

## 3. Parameters to Influence the Thermal Conductivity of Protein Polymers

In general, the Debye equation (Equation (1)) [55] is used to model the thermal conductivity κ of isotropic 3-D materials due to phonon transport [56]:

(1) $$\kappa =\frac{1}{3}C\upsilon l$$

where *C* is the volumetric heat capacity, *v* the speed of sound, and *l* the mean free path of the phonons, which is limited by point defects, scattering from sample boundaries, and phonon–phonon interactions [57]. The thermal conductivity of protein polymer materials can be governed by many factors such as crystallinity, molecular chain alignment, temperature, moisture, impurities, interfaces, and chemical bonding. Therefore, many recent studies have focused on manipulating thermal conductivity of polymer materials at the micro- and nano-scales [58,59,60,61,62,63,64,65,66,67,68,69,70]. 

### 3.1. Crystallinity and Crystal Alignment

**Figure 3:**
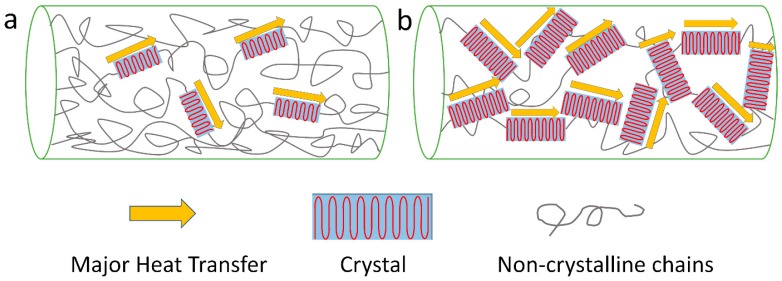
Effect of crystallinity on heat transfer: (**a**) structure of bulk polymer material with low crystallinity; (**b**) structures of polymer material with higher crystallinity.

**Figure 4:**
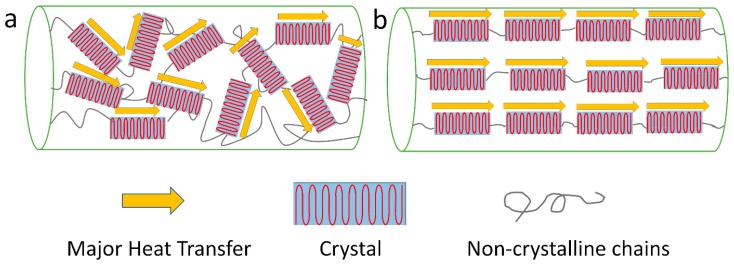
Effect of crystal alignment on heat transfer: (**a**) structure of bulk crystalized polymer with less crystal alignment; (**b**) structure of stretched crystalized polymer with good crystal orientation.

Many experiments have shown that polymers with high crystallinity have much higher *κ* values compared to that of their amorphous counterparts [5,7,71,72,73,74,75]. The amorphous structure decreases the mean free path of phonons, and disordered alignment will scatter phonons and decrease the speed of sound *v,* which can be seen in Figure 3 (effect of crystal content). Xu et al. reported that less crystalline structure and more random coils contributed to the relatively lower *κ* values of hexafluoroisopropanol (HFIP) film of *L. hesperus* [71]. A recent study by Tomko et al. found that tunable and reversible thermal conductivity of the hydrated tandem-repeat (TR) protein film can be achieved by altering its amorphous conformation or overall network topology. The κ values of the hydrated TR protein films is not only higher than that of the ambient TR protein films, but also related to the number of protein building block repeats [13]. On the other hand, Shen et al. found that the *κ* values of polyethylene nanofiber fabricated by a ultra-drawn method can reach as high as 104 W/m∙K [7]. After crystallization, the thermal conductivity of the polymer materials can be further improved, with an almost perfect crystal alignment orientation following the crystalline direction. A mechanism was proposed in Figure 4 to help understand this idea. It was also believed that the defect density of polymers would decrease after a crystallization process. 

### 3.2. Chain Orientation

**Figure 5:**
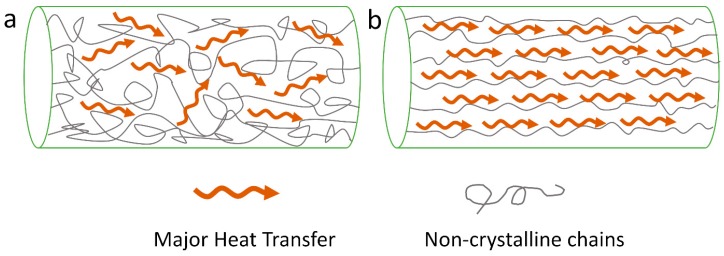
Effect of molecular chain alignment on heat transfer: (**a**) structure of bulk non-crystalized polymer with less chain alignment; (**b**) structure of stretched non-crystalized polymer with good chain orientation.

A high degree of chain orientation can help increase the *κ* values of polymers even with an amorphous conformation [76,77,78,79]. For example, progress has been made recently by Singh et al. [80] to improve the thermal conductivity of amorphous polythiophene using a nano-scale design method. The molecular chain orientation in the across-plane direction of polythiophene was significantly improved during electropolymerization through a nano-scale template (Figure 5b). The *κ* values of formed amorphous polythiophene reached up to 4.4 W/m∙K compared to 0.2 W/m∙K for the bulk polymer. The smaller the diameter of the nanofiber, the higher the degree of orientation and thermal conductivity. It was hypothesized that the enhancement of the chain orientation in polythiophene nanofibers increased the speed of sound in materials while decreasing the phonon scattering. This was subsequently confirmed by molecular simulation studies [81]. 

### 3.3. Composites

Another effective way to improve the *κ* values of polymers is to mix them with nano-structural materials that have high thermal conductivity such as carbon nanotubes, graphene, boron nitride nanosheets, nano-scale aluminum nitride, and copper nanoparticles [57,82,83,84,85,86,87,88,89,90,91,92,93,94]. Nanostructure fillers are not only used in elevating thermal conductivity but also in controlling electrical and mechanical properties of polymer composites. The *κ* values of a polymer composite can be directly controlled by the filler’s size, shape, volume fraction and distribution in the polymer matrix. 

For example, it was demonstrated that polyvinyl alcohol (PVA) incorporated with boron nitride nanotubes can be electrospun into composite mats with a much higher thermal conductivity than that of pure PVA mats [79]. The *κ* value of the film increased as the volume fraction and alignment degree of nano-filler increased. It is noted that nano-structural materials can provide a better thermal transport path which limits phonon scattering.

**Figure 6:**
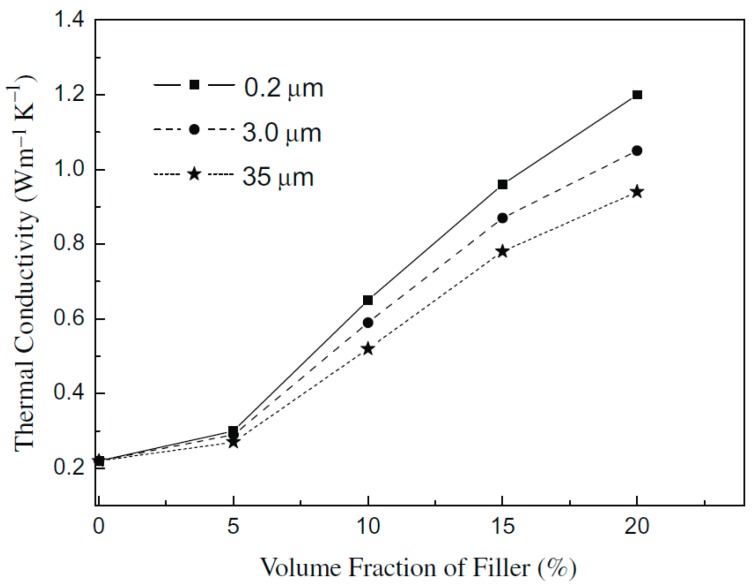
Impacts of filler diameter and volume fraction on thermal conductivity of composites. Reprinted with permission of Elsevier [95].

Due to the different phases in the polymer composites, which are phonon-based conductors, it is unavoidable that phonons would scatter at the interfaces. Therefore, the size and shape of the filler is important. A recent study reported that *κ* value of polymer composite decreased as the filler particle size increased when the filler volume fraction was above 5%, as shown in Figure 6 [95]. 

### 3.4. Other Parameters

Researchers have shown that the *κ* values of polymer nanocomposites can increase with the temperature [96,97]. Besides, it is also believed that the chemical process can help improve the interfacial bonding between the graphite nanoplatelets (GNPs) and polymer matrix, which also increased the *κ* values of the nanocomposites [96]. As reported by C. Cassignol et al., the *κ* values of polypyrrole (PPy) increased with the moisture content, especially from 8.5% to 13.5% [98]. Tomko et al. reported that the hydrated tandem-repeat (TR) protein films had an increased thermal conductivity compared to ambient state TR protein films [13]. Therefore, in addition to parameters such as crystallinity, chain orientation and fillers, the *κ* values of polymers also vary with the change of temperature, moisture and other factors [57,98,99]. Due to the anisotropic structure and microstructure of polymers, the *κ* values of polymer in the cross-plane direction or the in-plane direction can also be different [100,101,102,103,104].

## 4. Experimental Methods to Measure the Thermal Conductivity of Protein-Based Materials

*κ* is defined by the heat flow due to a temperature gradient. More precisely,

(2) $$\kappa =\frac{QL}{A\Delta T}$$

where *L* is the length, *A* the cross-sectional area and Δ*T* is the temperature difference across the ends of the sample. Fourier’s Law states that

(3) $$\frac{1}{A}\frac{dQ}{dt}=-\kappa \frac{dT}{dz}$$

where *dQ/dt* is the rate of heat flow along the *z* direction, and *dT*/*dz* the resulting thermal gradient. Many techniques have been developed in the last decades to measure the thermal conductivity of solids, nanoparticles and nanofluids [105,106,107,108,109]. Therefore, different kinds of experimental setups, as described below, have been made to measure the *κ* values of protein-based biopolymers [3,63,64,65,71,104,110,111].

### 4.1. Temperature-Modulated Differential Scanning Calorimetry Method

Differential scanning calorimetry (DSC) is a thermal analysis technology that can be used to measure various thermal and chemical properties of materials, such as glass transition temperature, decomposition temperature, melting point, crystallinity and oxidative stability. It is based on measuring the heat flow into or out of the specimen as a function of temperature or time [112,113,114]. Temperature-modulated differential scanning calorimetry (TMDSC) [115] divides the total heat flow into reversing part (heat capacity) and non-reversing part (kinetic). As a result, specific transition information, direct measurement of heat capacity and higher sensitivity can be obtained. TMDSC method was used to measure the *κ* values of silkworm cocoons in the thickness direction [65,72,115].

As reported by Zhang et al. [65], the measurement was under the protection of nitrogen gas, and the TMDSC had a temperature amplitude of ±1 °C and 60 s period. For a circular cylinder sample, the measured thermal conductivity *κ*_0_ is given by

(4) $$\kappa_{0}=\frac{8LC^{2}}{C_{p}md^{2}P}$$

where *d* is the diameter, *P* an experimental parameter, *m* the mass of the circular cylinder sample, *C* the apparent heat capacity, and *C_p_* the specific heat capacity of the sample, which can be measured directly by the TMDSC. Because of the heat loss through side areas of the circular cylinder sample, which resulted in the measurement discrepancy,

(5) $$\kappa =\frac{1}{2}\left[\kappa_{0}-2D+{\left(\kappa_{0}^{2}-4D\kappa_{0}\right)}^{\frac{1}{2}}\right]$$

where *D* is the thermal conductivity calibration constant, determined by *κ*_0_ and the *κ* value of the reference sample. 

**Figure 7:**
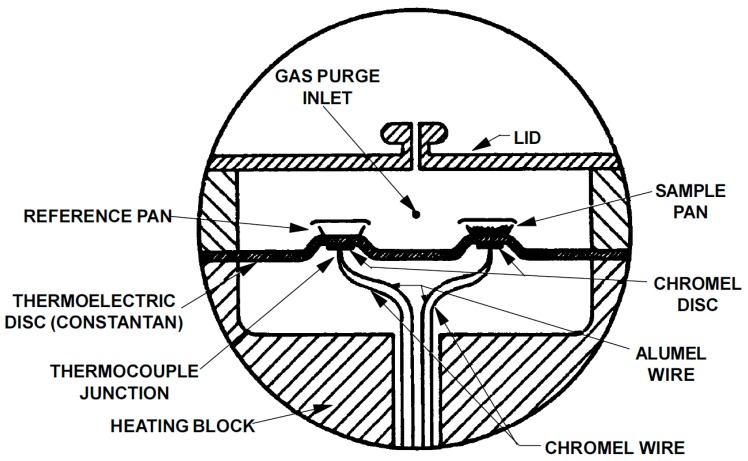
Schematic of the differential scanning calorimetry method. Reprinted with permission of Elsevier [116].

Figure 7 shows the schematic of TM-DSC method. In principle, the discrepancy from the purge gas may be reduced effectively with a low thermal conductivity purge gas, such as argon. Based on various assumptions [115,116,117,118,119], the accuracy of this method is around 3–4% from the values measured from other techniques. This method is limited to measure *κ* values in the range from 0.1 to 1.5 W/m∙K. However, one notes that recent studies also used conventional DSC to measure *κ* values.

### 4.2. 3-ω Method (Transient Hot Wire Method)

**Figure 8:**
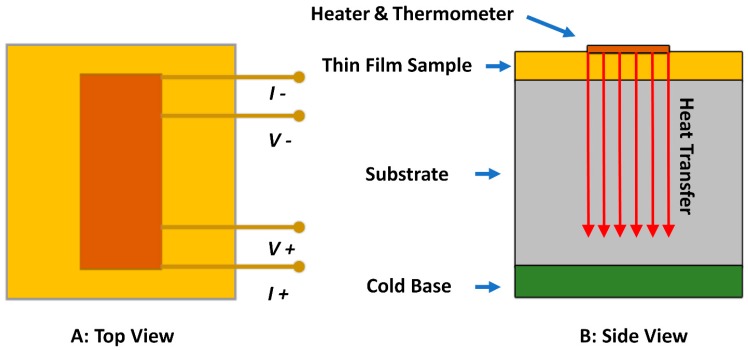
Experimental setup of the 3-*ω* method. A is the top view of the measurement setup, and B is the side view of the setup.

The 3-*ω* method is a technique that has been widely used to measure *κ* values of thin films for several decades [109,120]. Compared to contactless methods, it does not require expensive devices with a complicated setup. A typical experimental setup for the 3-*ω* method is shown in Figure 8. For this method, a narrow metal line is patterned on the surface of the film sample directly. Alternating current at angular frequency *ω* is applied to the metallic strip, and Joule heating is caused at a frequency of 2*ω*. In addition, the temperature-dependent resistance of the metal results in a voltage of third harmonic 3*ω*. As reported by Delan et al. [110], this method was used to test the thermal conductivity of a porous silk film in thickness direction. 

Even though the 3-*ω* method is a simple, fast, low-cost method with high accuracy, this technique is limited to electrically nonconductive materials [121]. Therefore, many extended/modified techniques have been developed recently to solve this problem, which also simplified the technique and increased the measurement accuracy [64,121]. 

### 4.3. Transient Electrothermal Technique (TET) Method 

**Figure 9:**
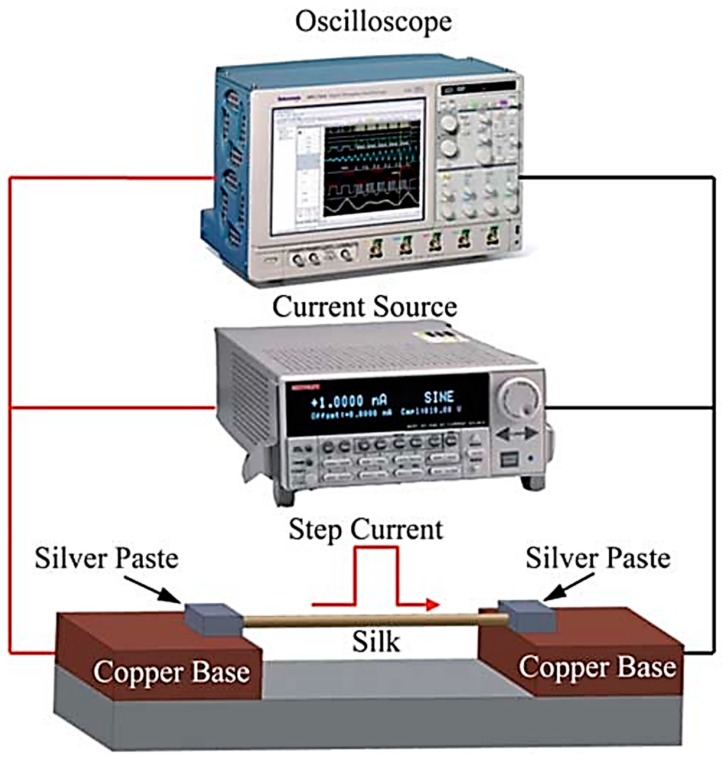
Schematic of the transient electrothermal technique (TET). Reprinted with permission of the Royal Society of Chemistry [64].

Based on the original 3-*ω* method, the transient electrothermal technique (TET) was developed by Liu et al. [64] to test the thermal conductivity of silk fiber in the axial direction. Figure 9 is a schematic of the TET method [64]. In order to keep the heat flow in one dimension, the length *L* of the silk fiber must be much longer than its diameter. A thin gold film is coated on the silk to make it electrically conductive. The two ends of the silk fiber are fixed on the copper base by silver paste with direct current (DC) fed through it. An oscilloscope is used to record the current and the induced voltage as a function of time. The measured thermal conductivity *κ*_0_ is given by

(6) $$\kappa_{0}=I^{2}RL/\left(12A\Delta T\right)$$

where *I* is the current, *R* the total resistance of silk fiber and gold film, *A* the total cross-sectional area of the silk fiber and gold film and Δ*T* the temperature difference. Δ*T* can be determined from the resistance change Δ*R* and the measured temperature coefficient of resistance *η*. Because of the gold film radiation, as well as the heat convection between the gold film and the surrounding gas, the value of κ of the silk fiber can be determined:

(7) $$\kappa =\kappa_{0}-\frac{L_{Lorenz}TL}{RA}$$

where *L_Lorenz_* is the Lorenz number. 

### 4.4. Photothermal Technique

**Figure 10:**
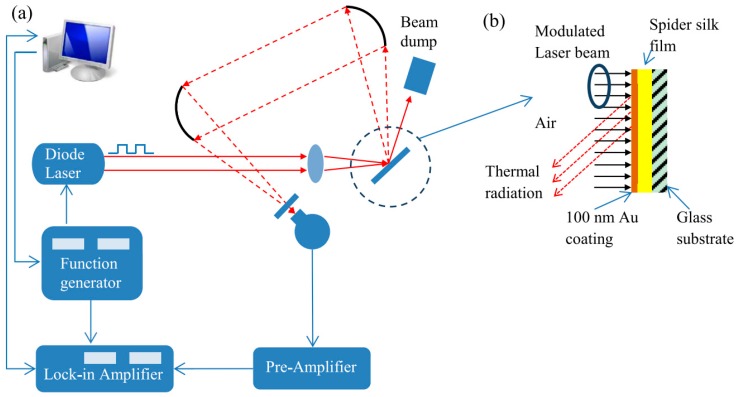
(**a**) Schematic for photothermal technique (PT) (**b**) Principle of photothermal technique (PT). Reprinted with permission of the Elsevier [71].

The photothermal technique (PT) method is an efficient way to measure thermal conductivity of carbon nanotubes, composite films and thin metal layers [122,123,124,125]. As reported by Xu et al., PT was used to measure the κ value of spider silk films [71]. A schematic of the PT method are shown in Figure 10. A thin gold film is coated onto the surface of the silk films. In this technique, the gold film is irradiated by a modulated-intensity laser. Due to the high κ value of the gold film, the temperature of the silk film changes periodically with a phase shift. The κ value of the silk film is determined by fitting the phase shift as a function of the modulation frequency of the laser. Alternatively, the κ value can be calculated from fitting the amplitude of thermal radiation from the gold film although this generally has less accuracy.

## 5. Thermal Conductivity of Different Types of Protein-Based Materials

There are many studies discussing the thermal conductivity of silkworm silk. Under relaxed conditions, the thermal conductivity of silkworm silk in the axial direction is 0.042 W/m∙K [64]. Under tension, the thermal conductivity increases. At 68% elongation, silkworm silk achieves its highest thermal conductivity of 13.1 W/m∙K [64]. Beyond that elongation point, its κ value and thermal diffusivity decrease rapidly with strain.

There has been a report by Huang et al. that spider silk a very high κ value of 340 W/m∙K [63]. They reported that the κ value increased to 415.9 W/m∙K under a strain of 19.7%. However, this claim is not universally accepted. The measured κ value and thermal diffusivity of *Nephila clavipes* spider silk reported by Xing et al. was 1.2 W/m∙K and 6 × 10^−7^ m^2^/s, respectively [37]. Xing and coworkers explained the thermal conductivity difference may be attributed to the vacuum level and heat transfer analysis method. Results published by Fuente et al. [39] claimed that the thermal diffusivity of spider silk is around 2 × 10^−7^ m^2^/s, which is 400 times lower than the value reported by Huang et al. [63]. Due to the extremely thin diameter of spider fibers, it is challenging to get accurate thermal conductivity and thermal diffusivity values, and more refined techniques need to be developed.

The thermal conductivity values of collagens and keratins were reported before. It showed that the κ value of sheep collagens is a linear function of temperature between 25 and 50 °C, and the values were around 0.53 W/m∙K [103]. The thermal conductivity of keratins has been measured as earlier as 1945, and the κ value of wool fibers in the diameter direction is around 4.62 × 10^−4^ W/m∙K, confirming their excellent thermal insulation properties [104].

## 6. Thermal Conductivity Differences between 1-D Fibers and 2-D Films

As compared to silk films, the structure of silk fiber is much simpler and can be easily characterized and manipulated. Liu et al. reported that the κ value of silkworm silk fiber in the axial direction ranges from 0.54–6.53 W/m∙K. However, when the silk fiber was stretched, the κ value of the stretched silk fiber increased, and at an elongation of 63.8%, the κ value was up to 13.1 W/m∙K. However, thermal conductivity of both silkworm silk films and spider silk films in the thickness direction is only in a range from 0.15–1 W/m∙K [3,65,71,110].

In these studies, Raman spectroscopy, scanning electron microscopy [126], DSC, and infrared spectroscopy (FTIR) were used to characterize the structure of the films and the single fibers. As noted above, higher crystallinity and molecular chain alignment result in a higher thermal conductivity. When a single fiber is strained, the crystallinity increases, which means the amorphous structure transforms into β-pleated sheets. As for silk films, due to their uncontrolled process of synthesis, lower degree of alignment, lower crystallinity, and higher porosity, they have relatively low thermal conductivity in the cross-plane direction.

## 7. Conclusions and Future Development

Extraordinary mechanical properties and biocompatibility of protein-based materials, such as silks, collagens and keratins, outperform many synthetic polymer materials. Because of their excellent chemical and physical properties, protein-based materials and their composites with extremely high or low thermal conductivity may have a great potential in new technology. Although extremely high thermal conductivity of a single silk fiber has been claimed, the thermal conductivity of protein-based materials, such as silk films, are still relatively low. Composites with high thermal conductivity nano-additives, such as carbon nanotubes and graphene, may lead to protein-based materials with high thermal conductivity in the future. Nonetheless, it is important to develop a creative method to prepare protein-based biopolymer materials with high crystallinity and chain alignment along with fewer defects to continue progress toward highly thermally conductive protein-based materials.

## Figures and Tables

**Table 1:** Thermal conductivity values of common polymers and metals at room temperature [7,8,9,10,11,12,13].

Material	Thermal Conductivity (W/m∙K)
Low density polyethylene (LDPE)	0.3
High density polyethylene (HDPE)	0.44
Polycarbonate	0.22
Polyvinyl chloride (PVC)	0.19
Nylon-6 (PA6)	0.25
Polythiophene nanofibers (amorphous)	~4.4
Polyethylene nanofibers	~104
Silkworm silk (axial direction)	~6.53
Flax fiber	0.1187
Squid protein	0.3–1.3
Silk/wool hybrid	0.000397–0.000663
Human skin	0.23–0.488
Aluminum	235
Copper	400
Nickel	158
Gold	345
Aluminum	235
Diamond	1000
